# Human osteoarthritis knee joint synovial fluids cleave and activate Proteinase-Activated Receptor (PAR) mediated signaling

**DOI:** 10.1038/s41598-023-28068-3

**Published:** 2023-01-20

**Authors:** Arundhasa Chandrabalan, Andrew Firth, Robert B. Litchfield, C. Thomas Appleton, Alan Getgood, Rithwik Ramachandran

**Affiliations:** 1 Department of Physiology and Pharmacology, Bone and Joint Institute, Schulich School of Medicine and Dentistry, University of Western Ontario, London, ON N6A 5C1 Canada; 2 Division of Orthopedic Surgery, Bone and Joint Institute, Fowler Kennedy Sport Medicine Clinic, Schulich School of Medicine and Dentistry, University of Western Ontario, London, ON Canada; 3 Department of Medicine, Bone and Joint Institute, Schulich School of Medicine and Dentistry, The Dr. Sandy Kirkley Centre for Musculoskeletal Research, London, ON Canada

**Keywords:** Osteoarthritis, Receptor pharmacology

## Abstract

Osteoarthritis (OA) is the most prevalent joint disorder with increasing worldwide incidence. Mechanistic insights into OA pathophysiology are evolving and there are currently no disease-modifying OA drugs. An increase in protease activity is linked to progressive degradation of the cartilage in OA. Proteases also trigger inflammation through a family of G protein-coupled receptors (GPCRs) called the Proteinase-Activated Receptors (PARs). PAR signaling can trigger pro-inflammatory responses and targeting PARs is proposed as a therapeutic approach in OA. Several enzymes can cleave the PAR N-terminus, but the endogenous protease activators of PARs in OA remain unclear. Here we characterized PAR activating enzymes in knee joint synovial fluids from OA patients and healthy donors using genetically encoded PAR biosensor expressing cells. Calcium signaling assays were performed to examine receptor activation. The class and type of enzymes cleaving the PARs was further characterized using protease inhibitors and fluorogenic substrates. We find that PAR1, PAR2 and PAR4 activating enzymes are present in knee joint synovial fluids from healthy controls and OA patients. Compared to healthy controls, PAR1 activating enzymes are elevated in OA synovial fluids while PAR4 activating enzyme levels are decreased. Using enzyme class and type selective inhibitors and fluorogenic substrates we find that multiple PAR activating enzymes are present in OA joint fluids and identify serine proteinases (thrombin and trypsin-like) and matrix metalloproteinases as the major classes of PAR activating enzymes in the OA synovial fluids. Synovial fluid driven increase in calcium signaling was significantly reduced in cells treated with PAR1 and PAR2 antagonists, but not in PAR4 antagonist treated cells. OA associated elevation of PAR1 cleavage suggests that targeting this receptor may be beneficial in the treatment of OA.

## Introduction

Osteoarthritis (OA) is the most common form of arthritis and a major health care burden with an estimated 250 million people currently affected^1–3^. In the context of this substantial global burden, most patients with OA receive symptomatic treatment alone given the paucity of effective disease-modifying agents^2^. The difficulty in identifying effective therapeutics stems in large part from the complex nature of this chronic disease, with many modifiable and non-modifiable risk factors including age, obesity, sex, trauma, physical activity, genetics and numerous environmental influences implicated in OA^3^. Irrespective of the etiology, a key feature of OA is the progressive degradation of the cartilage with an increase in proteolytic enzyme activity linked to this damage^4,5^. Synovial inflammation^6^ and bone remodelling^7^ are also important pathophysiological events in OA.

The role of proteolytic enzymes as important regulators of damage in OA has been long recognized. The matrix metalloproteinases (MMPs) in particular have received much attention with their collagenolytic activity playing an important role in OA pathology^4^. The enzymatic activity of MMPs and aggrecanases weaken the cartilage matrix, making it more susceptible to mechanical disruption during joint loading and movement. Other enzymes such as the cysteine proteinase cathepsin-K can also degrade proteins in the cartilage and bone extracellular matrix (ECM)^5^. In addition to the key matrix-degrading MMPs and cysteine proteinases, serine proteinases such as matriptase, coagulation cascade enzymes, kallikrein-related peptidases and neutrophil enzymes, derived from both structural and immune cells in the joints, contribute to ECM remodelling, tissue healing, pain, inflammation and immunity, and as important regulators of OA pathogenesis^8^.

A key mechanism by which these proteolytic enzymes perpetuate pathological conditions in the joint is by activating a four-member family of G protein-coupled receptors (GPCRs) called the Proteinase-Activated Receptors (PARs, PAR1-4)^9^. Expression of PAR1^10–13^, PAR2^14–18^, PAR3^19^ and PAR4^20–22^ are documented in cells of the synovium, cartilage, bone and neurons^8,23^. The role of PAR3 in physiology and pathophysiology however remains poorly understood since this receptor cannot signal independently^24^. PAR activation is generally considered to be proinflammatory^17,25,26^ and proalgesic^21^, though roles of specific receptors and associated signaling events remain unclear with model-specific and mechanistic differences in protection afforded by receptor deletion noted^27,28^.

A significant challenge in understanding the role of PARs in joint disease relates to the variety of enzymes that can activate these receptors^9,29^. PAR1 is classically described as thrombin activated receptor, but certain MMPs^30,31^ and neutrophil-derived enzymes including elastase, proteinase-3^32^ and cathepsin-G^33^ also cleave PAR1. PAR2 is similarly cleaved by a number of trypsin-like enzymes such as trypsin^34,35^, matriptase^36^, mast cell tryptase^37^, neutrophil-derived enzymes^38^ and cathepsins^39^. PAR4 can be similarly proteolytically processed by thrombin, trypsin and neutrophil cathepsin-G^40,41^. Irrespective of the proteinases involved, PAR activation requires enzymatic cleavage of extracellular receptor N-terminus to unmask a motif called the tethered-ligand, which then binds intramolecularly and activates the receptors. Interestingly, different enzymes cleave the PARs at different positions on the receptor N-terminus, leading to different tethered-ligands being generated, and consequent activation of different or biased signaling cascades^42^. PAR activation can also be silenced or disarmed in some instances where proteolytic cleavage removes tethered ligand sequences that activate the receptor^33,43–45^. The diversity of PAR regulating enzymes present in the joint spaces is poorly understood and this remains a significant challenge in understanding PAR signaling in OA. Compounding this problem further, there exist species-specific differences in the complement of both proteinases and the PAR receptors^29,46^. In this context, previous studies have not directly examined PAR cleavage and activation by proteinases present in human osteoarthritic knee joints. In the current study, we have sought to address this using novel genetically encoded PAR biosensors to examine PAR1, PAR2 and PAR4 activating synovial fluid enzymes in patients with knee OA.

## Materials and methods

All studies with human samples were approved by the Western University Health Sciences Research Ethics Board and were in accordance with the Declaration of Helsinki. All research complied with relevant guidelines and informed consent was obtained from all participants. Healthy donor knee synovial fluids were obtained from Rheumatology Centre (C.T.A) at St. Joseph’s Health Care London, ON, Canada (ethics ID # REB 109255). Synovial fluid samples from OA patients undergoing realignment osteotomy were obtained from the Fowler Kennedy Sport Medicine Clinic and University Hospital at London Health Sciences Centre, London, ON, Canada (ethics ID # REB 108039). At surgery, OA patient synovial fluids from knee joints were collected by an orthopedic surgeon (A.G or R.B.L) prior to commencing arthroscopy and proximal tibial osteotomy. All samples were immediately placed on ice and transferred to the laboratory, centrifuged at 10,000 × *g* for 1 min, aliquoted and frozen at − 80 °C. All joint fluids were tested for presence of any blood with the Fecal Occult Blood Test kit (detection level: 0.6 mg Hb/g, Immunostics Hema-Screen) and samples showing presence of blood were excluded from further analysis.

Thrombin (human plasma, specific activity ≥ 2800 NIH units/mg protein, Calbiochem-EMD Millipore) and trypsin (porcine pancreas, Type IX-S, 13,000–20,000 BAEE units/mg protein, Sigma-Aldrich) stock solutions were made in 25 mM 4-(2-hydroxyethyl)-1-piperazine ethanesulfonic acid (HEPES, Fisher Scientific). The thrombin-selective inhibitor PPACK.2HCl and MMP Inhibitor V (ONO-4817) were obtained from Calbiochem (Millipore Sigma). The broad-spectrum MMP inhibitor batimastat (BB-94, ≥ 98%) was from Sigma-Aldrich. Soybean trypsin inhibitor (STI) was from ThermoFisher Scientific or Millipore Sigma. The fluorogenic substrates, Bz-Phe-Val-Arg-AMC.HCl (Thrombin substrate III) and Boc-Gln-Ala-Arg-AMC.HCl (Trypsin substrate) were from Bachem, Suc-Ala-Ala-Pro-Phe-AMC (Chymotrypsin substrate II) and Z-Gly-Gly-Arg-AMC.HCl (Urokinase substrate III) were from Calbiochem, and MCA-Lys-Pro-Leu-Gly-Leu-Dpa(DNP)-Ala-Arg-NH_2_ (MMP substrate FS-6) was from Sigma-Aldrich. The stock solutions of the enzyme inhibitors and fluorogenic substrates were prepared according to manufacturer's instructions. PAR agonist peptides TFLLR-NH_2_ (PAR1), SLIGRL-NH_2_ (PAR2) and AYPGKF-NH_2_ (PAR4) were from GenScript Biotech, and stock solutions were made in 25 mM HEPES. PAR1 selective antagonist Vorapaxar was purchased from Adooq Bioscience, PAR2 antagonist AZ3451 from Sigma, and PAR4 antagonist BMS-986120 from Cayman Chemical. The stock solutions of the PAR antagonists were prepared in dimethyl sulfoxide (DMSO, BioShop). All samples were diluted to appropriate working concentrations in Hanks' Balanced Salt Solution containing CaCl_2_ and MgCl_2_ (HBSS, Gibco ThermoFisher Scientific). Fluorogenic substrates were diluted in Tris-NP40-calcium buffer [50 mM Tris.HCl (Fisher BioReagents) pH 8, 0.2% Nonidet P-40 Substitute (Roche) and 1.5 mM CaCl_2_ (Fisher BioReagents)].

### Patient inclusion criteria

Patients with a primary diagnosis of symptomatic knee OA undergoing a coronal plane alignment correction by opening wedge proximal tibial osteotomy were included. Patients undergoing concomitant procedures such as ligament reconstruction, meniscal transplantation or cartilage restoration were excluded. Patients with inflammatory arthropathy or past history of joint infection were also excluded. All patients completed a radiological examination including weight bearing anteroposterior, 45-degree flexed posteroanterior, lateral and standing hip-knee-ankle alignment views. The amount of radiographic knee OA was graded independently by two orthopedic surgical fellows on a scale of grade 0 (none), 1 (doubtful), 2 (mild), 3 (moderate), and 4 (severe), according to the Kellgren and Lawrence (KL) classification system^47^. Absence of knee symptoms and radiographically confirmed KL 0 was considered healthy. Patients also completed Knee Injury and Osteoarthritis Outcome Score (KOOS)^48^, the Western Ontario and McMaster Universities Arthritis Index (WOMAC)^49^, and Visual Analog Scale (VAS) pain score^50^ questionnaires at baseline prior to surgery. The total KOOS is a mean percentage score of the five subscales encompassing pain, symptoms, activities in daily living function (ADL), sport and recreation function (Sport/Rec), and quality of life (QOL), score mean^48,51^. The total WOMAC is a mean percentage score of the three subscales encompassing pain, stiffness, and physical function^49^. In addition, each subscale score was calculated independently and transformed to a percentage score. Each score was converted to a percentage score by using the formula below, where KOOS and WOMAC transformed score of 100% represents no problems and 0% indicates extreme problems. VAS pain score rates the patient’s pain at rest and during activity (move) on average over the past week, and the scale ranges from 0 (no pain) to 10 (worst pain possible). The mark placed along the scale (0–10 cm) by the patient is measured as the level of pain for each situation^50^.

$$Transformed\,score = 100\% - \left( {\frac{actual\,\,raw\,\,score}{{maximum\,score}} \times 100\% } \right).$$

### Cell culture

Chinese Hamster Ovary (CHO-K1, Sigma) cells were cultured in Ham's F-12 Nutrient Mix supplemented with 1 mM l-glutamine, 100 U ml^−1^ penicillin, 100 µg ml^−1^ streptomycin, 1 mM sodium pyruvate, and 10% v/v heat-inactivated Fetal Bovine Serum (FBS, Gibco ThermoFisher Scientific). CHO cells stably transfected with the PAR biosensors cloned into pcDNA3.1(+) were cultured in complete F-12 medium with 600 µg ml^−1^ geneticin selective antibiotic (G418 Sulfate, Gibco ThermoFisher Scientific). The cells were grown in a T75 cell culture flask (Nunc) in a humidified cell culture incubator with 5% CO_2_ at 37 °C. Cells at ~ 80–90% confluency were detached with phosphate-buffered saline (PBS, Gibco ThermoFisher Scientific) solution supplemented with 1 mM EDTA (Fisher Scientific), centrifuged at 180 × *g* for 5 min, and sub-cultured as appropriate.

Human embryonic kidney (HEK)-293 cells (ATCC) stably transfected with PAR4-eYFP-pcDNA3.1(+)^52^ were maintained in Dulbecco’s Modified Eagle’s Medium (DMEM, Gibco ThermoFisher Scientific) supplemented with 1 mM sodium pyruvate, 100 U ml^−1^ penicillin, 100 µg ml^−1^ streptomycin, 2 mM l-glutamine, 10% v/v FBS, and 600 µg ml^−1^ G418. Cells at ~ 80–90% confluency were dissociated with trypsin–EDTA (0.25%, Gibco ThermoFisher Scientific), centrifuged at 180 × *g* for 5 min, and sub-cultured as appropriate.

### Biosensor cloning and stable transfection

Human PAR2 with N-terminal nano-luciferase (nLuc, Promega) and C-terminal enhanced Yellow Fluorescent Protein (eYFP) tagged constructs have been previously described^53^. N-Terminal nLuc tagged human PAR1 and PAR4 constructs were similarly constructed by generating restriction enzyme sites for BspE1 and BamH1 by site-directed mutagenesis (QuickChange, Agilent technologies) and inserting nLuc in previously described eYFP tagged receptor constructs^38,52^. nLuc was located between the residues Glu^30^Ser^31^ in PAR1, Gly^28^Thr^29^ in PAR2, and Pro^23^Ser^24^ in PAR4. Fidelity of all constructs was verified by direct sequencing (London Regional Genomics Centre, Robarts Research Institute). CHO-K1 cells were stably transfected with the nLuc and eYFP tagged hPAR1, hPAR2, or hPAR4-pcDNA3.1( +) constructs by electroporation (Super Electroporator NEPA21 Type II, Nepa Gene) with 3 µg plasmid DNA and 1 × 10^6^ cells in 100 µl Opti-MEM (1 ×) Reduced Serum Medium (Gibco ThermoFisher Scientific). The electroporated cells were cultured in a non-selective complete medium in a 100 mm × 20 mm cell culture dish (Falcon, Corning) for 48–72 h. The cells were subsequently maintained in G418 selective medium in a T75 flask for 7–14 days. G418-resistant cells expressing the construct (nLuc-hPAR1/2/4-eYFP) were clonally sorted by flow cytometry (FACSAriaIII, London Regional Flow Cytometry Facility, Robarts Research Institute) and expanded in G418 selective medium. The stably transfected reporter cell lines were characterized with a known hPAR agonist, thrombin on nLuc-hPAR1-eYFP, trypsin on nLuc-hPAR2-eYFP, and both thrombin and trypsin on nLuc-hPAR4-eYFP, using the luciferase assay technique described below. As a negative control, luminescence levels in the parental CHO-K1 cells treated with PAR agonists thrombin and trypsin were also assessed.

### Luciferase assay

The presence of active PAR cleaving enzymes was measured by monitoring release of the N-terminal nLuc fusion tag. The nLuc-hPAR1/2/4-eYFP-CHO reporter cells were plated either in a 24-well or 96-well cell culture plates (polystyrene, flat-clear bottom, Nunclon Delta, Nunc, ThermoFisher Scientific) at a cell density of 1 × 10^4^ cells per well in complete F-12 medium and cultured for 48 h. The cells were rinsed with HBSS (3 × 100 µl) and incubated with 100 µl HBSS at 37 °C for 15 min. 50 µl of cell supernatant from each well was transferred into a 96-microwell white plate (polystyrene, Nunclon Delta, Nunc, ThermoFisher Scientific) and this served as a measure of basal luminescence levels in each well. The cells were then incubated with 50 µl of test samples, recombinant enzymes or controls at 37 °C for 15 min. 50 µl of cell supernatant from treated wells were transferred to a white plate. The Nano-Glo Luciferase Assay Substrate furimazine (2 µl ml^−1^, Promega) was added and the luminescence was measured on a luminometer (Mithras LB 940 Berthold Technologies plate reader, measurement time: 1 s per well).

### Fluorogenic substrate assay

Proteinase activity in synovial fluids was measured using peptide-based fluorogenic substrates. The synovial fluid samples (10%) were either untreated or pretreated with enzyme inhibitors, PPACK (1 µM), STI (1 mg ml^−1^), BB-94 (10 µM) or ONO-4817 (10 µM) for 30 min at room temperature. Substrate cleavage was measured in a clear bottom black 96-well microplate (Greiner Bio-One) with thrombin, trypsin, chymotrypsin, and urokinase substrates (100 µM final concentration in 60 µl), and MMP substrate (25 µM final concentration in 60 µl). Immediately following the addition of the substrates, the fluorescence was measured every minute for 10–60 min on a fluorometer. The substrates containing fluorescent 7-amino-4-methylcoumarin (AMC) were read on a PerkinElmer Victor plate reader (λ_Ex:Em_ 355:460 nm; measurement time: 0.1 s per well; lamp energy: 40,000), and the MMP substrate containing the fluorophore (7-methoxy-coumarin-4-yl)acetyl (MCA) was read on a Cytation5 BioTek plate reader (fluorescence endpoint, λ_Ex:Em_ 325/20:390/20 nm; measurement time: 0.1 s per well; gain: extended; lamp energy: high, extended dynamic range).

### Calcium signaling assay

Synovial fluids activation of PAR mediated calcium signaling was assessed in HEK293 cells that endogenously express PAR1 and PAR2, and stably transfected PAR4. PAR activated Gα_q/11_-mediated calcium signaling was measured as described previously^54^ with some modifications. HEK293 cells were seeded at a density of 2 × 10^4^ cells per well in a poly-D-lysine (Sigma) coated^54^ 96-well black cell culture microplate (Optical Bottom, Polystyrene, Thermo Scientific) and cultured for 24 h. The cells were rinsed with PBS (2 × 100 µl per well) and incubated with Fluo-4 NW calcium indicator (50 µl per well, Life Technologies) for 30 min at 37 °C and for an additional 15 min at room temperature in the dark. The change in fluorescence as an indicator of change in intracellular calcium levels was measured on a FlexStation3 (Molecular Devices) microplate reader. Data was recorded for 20 s to obtain baseline fluorescence levels prior to addition of agonist and for further 160 s. To measure antagonism, the cells were pre-treated with receptor antagonists for 30 min at room temperature. Calcium ionophore (A23187, 6 µM, Sigma-Aldrich) was used as a control in all experiments to determine the maximum response in each well.

### Graphical and statistical analyses

The luminescence measurements in the luciferase assay were normalized by subtracting the basal luminescence in HBSS treated samples. The concentration-effect curves of the standard enzymes were plotted and analyzed using the dose–response stimulation three parameters model with a non-linear regression curve fit and the logEC_50_ values with standard error of the mean (SEM) were obtained on GraphPad Prism 8. Each data point on the concentration-effect curve corresponds to the mean of at least three independent experiments (*N* = 3–4), performed either in duplicates (*n* = 6) or triplicates (*n* = 9), with their SEM. The normalized luminescence measurements of the test samples were calculated as a percentage of the maximum response of the standard agonist, thrombin (3 NIH U ml^−1^; ≈ 30 nM) for PAR1 and PAR4, and trypsin (100 nM; ≈ 50 BAEE U ml^−1^) for PAR2. The data represents the mean of at least three independent experiments (*N* = 3–5), performed in duplicates (*n* = 2), with their SEM. The fluorescence measurements from the fluorogenic substrate assay were normalized by subtracting the fluorescence values obtained with the substrate alone (blank). The initial slope of the kinetic curves (10 min measurements from the addition of thrombin and trypsin substrates, and 60 min measurements from the addition of MMP substrate) were calculated on GraphPad Prism 8 as a measure of the enzymatic activity in synovial fluids. The data represents the mean of three independent experiments (*N* = 3), performed in duplicates (*n* = 2), with their SEM. In calcium signaling, the fluorescence intensities were normalized by subtracting the baseline signal, and each antagonist treated synovial fluid response was calculated as a percentage of the corresponding untreated synovial fluid response. Data are presented as the mean of three independent experiments (*N* = 3), performed in duplicates (*n* = 2), with their SEM. Mann–Whitney U test with 95% confidence intervals (CI) was utilized to compare any statistical significance between two groups, and one-way ANOVA Kruskal–Wallis test was utilized to assess differences between multiple groups, at *p* < 0.05. Cohen’s kappa (*κ*) was used to determine the inter-rater agreement between the two clinicians when grading KL knee OA. Agreement was interpreted using the scale *κ* < 0.20: slight agreement, *κ* = 0.21–0.40: fair agreement, *κ* = 0.41–0.60: moderate agreement, *κ* = 0.61–0.80: substantial agreement, and *κ* = 0.81–1.0: almost perfect agreement^55^. Pearson correlation coefficient analysis on GraphPad Prism 8 was used to determine the correlation between PAR activity and subscales of KOOS/WOMAC/VAS scores. Correlation coefficient was interpreted based on the reported scale^56^ r =  ± 1.00 to ± 0.90: a very strong correlation, r =  ± 0.89 to ± 0.70: a strong correlation, r =  ± 0.69 to ± 0.40: a moderate correlation, r =  ± 0.39 to ± 0.10: a weak correlation, and r =  ± 0.10 to ± 0.00: negligible correlation.

## Results

### Patient characteristics

Demographics and radiographic data of the four healthy donors (H1-H4) and twenty-five OA patients (1–25) are presented in Table 1. The degree of radiographic knee OA was graded on a scale of grade 0 (none), 1 (doubtful), 2 (mild), 3 (moderate) and 4 (severe) according to the KL classification system^47^. Absence of knee symptoms and radiographically confirmed KL 0 was considered healthy. Fifteen male and ten female patients ranging from doubtful OA (KL grade 1) to moderate OA (KL grade 3) at the time of sample collection, and one male and three female healthy donors with no knee symptoms or radiographic features of OA (KL grade 0) were included in the study. The two clinicians demonstrated moderate inter-rater agreement when grading KL knee OA (*κ* = 0.49, 95% CI 0.23 to 0.79). The median age ± SEM of the OA patients studied here was 49 ± 9 years and 25 ± 16 years for healthy donors. There was no statistically significant difference in age between healthy and OA patient groups. Twenty of the twenty-five OA patients in this study fit the criteria for being overweight (BMI 25.0–29.9 kg m^−2^) or obese (BMI > 29.9 kg m^−2^). Three of the four healthy donors were in the normal weight (BMI 18.5–24.9 kg m^−2^) category, and one was overweight. Patient reported KOOS, WOMAC and VAS scores of all OA patients are presented in the supplementary data Table S1.

**Table 1:** Patient demographics and radiographic data.

Patient	Sex	Age (years)	BMI (kg m^−2^)	KL grade	Patient	Sex	Age (years)	BMI (kg m^−2^)	KL grade
**H1**	M	23	25.2	0	**12**	M	52	38.9	2
**H2**	F	22	24.6	0	**13**	F	42	37.8	2
**H3**	F	27	22.2	0	**14**	F	43	30.4	2
**H4**	F	54	22.1	0	**15**	F	44	24.4	2
**1**	M	24	24.0	1	**16**	F	53	35.1	2
**2**	M	38	29.5	1	**17**	F	55	34.8	2
**3**	M	46	34.3	1	**18**	M	49	24.8	3
**4**	M	49	32.2	1	**19**	M	50	29.4	3
**5**	M	53	34.3	1	**20**	M	52	34.1	3
**6**	F	27	30.9	1	**21**	M	54	25.9	n.a
**7**	F	28	49.3	1	**22**	M	58	24.4	3
**8**	F	43	26.7	1	**23**	M	61	23.8	3
**9**	M	35	28.9	2	**24**	F	50	31.8	3
**10**	M	37	40.6	2	**25**	F	56	33.3	3
**11**	M	48	29.4	2					

*H* healthy donor, *M* male, *F* female, *BMI* body mass index, *KL* Kellgren and Lawrence system of knee OA classification, *n.a.* not available.

### Characterization of PAR biosensor expressing cell lines

**Figure 1:**
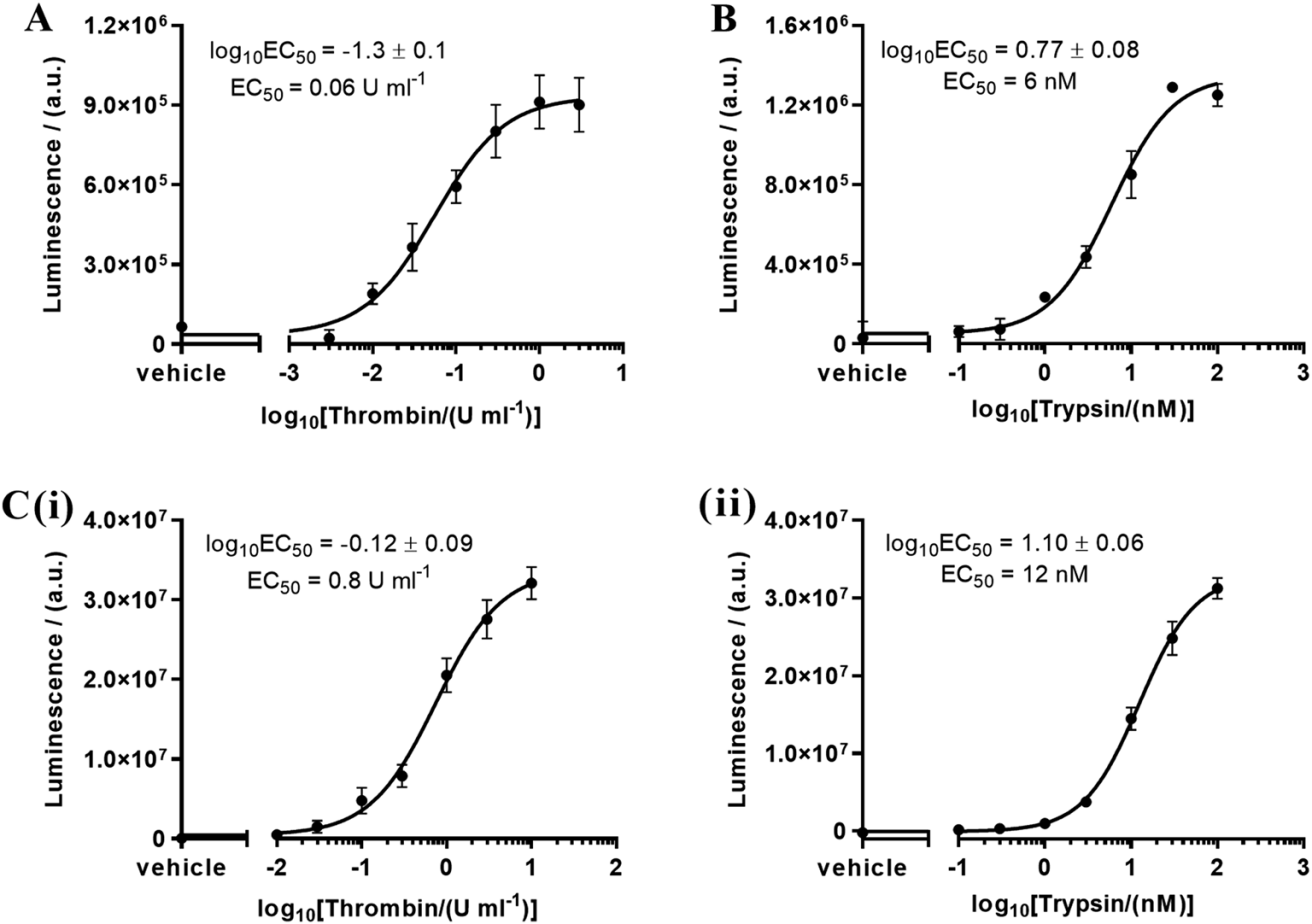
Characterization of PAR biosensor expressing reporter cell lines, (**A**) nLuc-hPAR1-eYFP-CHO response to thrombin (0.003–3 U ml^−1^), (**B**) nLuc-hPAR2-eYFP-CHO response to trypsin (0.1–100 nM), and (C) nLuc-hPAR4-eYFP-CHO response to (**i**) thrombin (0.01–10 U ml^−1^) and (**ii**) trypsin (0.1–100 nM). Each data point on the concentration-effect curve represents the mean ± SEM of at least three independent experiments (*N* = 3–4) performed in triplicate.

PAR biosensor expressing reporter cell lines nLuc-hPAR1-eYFP-CHO, nLuc-hPAR2-eYFP-CHO and nLuc-hPAR4-eYFP-CHO were characterized by monitoring responses to standard PAR activating enzymes, thrombin and/or trypsin (Fig. 1). The cleavage of PAR1, PAR2 and PAR4 by the canonical activator enzymes was assessed by measuring the release of the N-terminal nLuc tag. Luminescence levels were assessed in the presence of the nano-luciferase substrate furimazine. The EC_50_ obtained for thrombin cleavage of PAR1 (0.06 U ml^−1^, Fig. 1A), trypsin cleavage of PAR2 (6 nM, Fig. 1B), and thrombin (0.8 U ml^−1^, Fig. 1C(i)) and trypsin (12 nM, Fig. 1C(ii)) cleavage of PAR4 were consistent with the enzyme concentrations eliciting PAR signaling in previously published work^32,38,57–60^. As expected, parental CHO-K1 cells treated with thrombin or trypsin did not show any luminescence signal.

### PAR1, PAR2 and PAR4 cleavage by enzymes in synovial fluids

**Figure 2:**
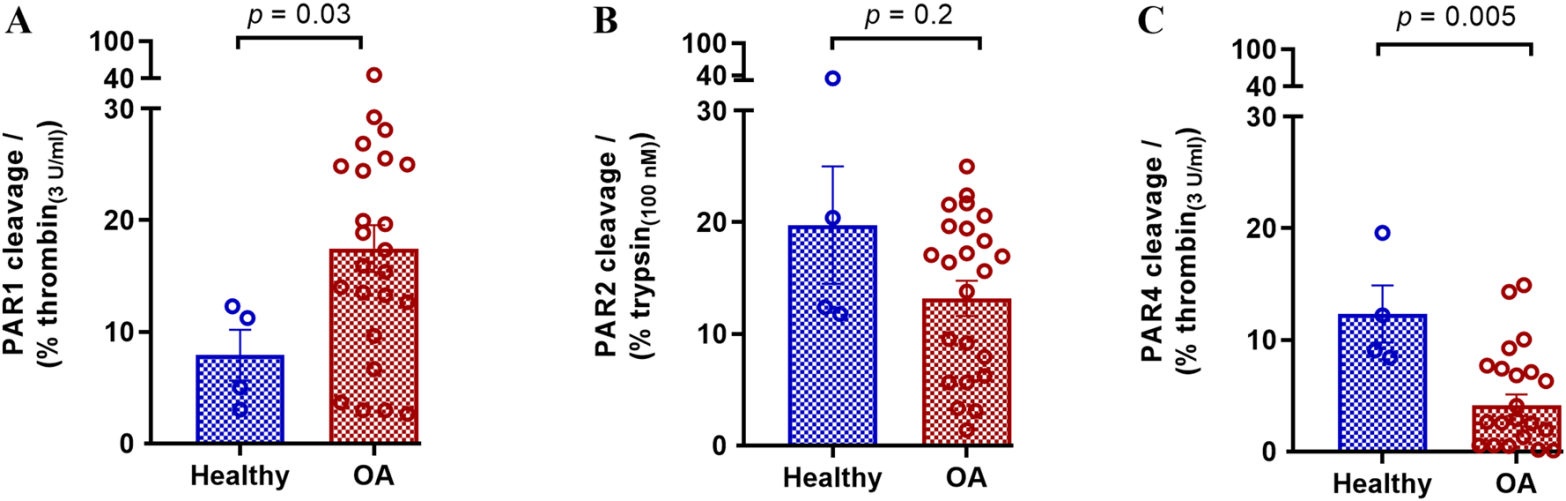
Cleavage of PARs by enzymes in human OA knee joint synovial fluids. Healthy and OA patient synovial fluids (10%) were applied to nLuc-hPAR-eYFP-CHO cells and release of the nLuc tag into the culture supernatants was monitored as an index of receptor N-terminus cleavage. (**A**) Cleavage of PAR1 by synovial fluids as a % of 3 U ml^−1^ thrombin cleavage, (**B**) Cleavage of PAR2 by synovial fluids as a % of 100 nM trypsin cleavage, and (**C**) Cleavage of PAR4 as a % of 3 U ml^−1^ thrombin cleavage. Histogram represents the mean cleavage ± SEM (*N* = 3–5) performed in triplicate. Mann–Whitney U test was utilized to assess differences between groups. *p* < 0.05 was considered statistically significant.

Cleavage of PAR1, PAR2 and PAR4 was assessed with the four healthy (**H1**–**H4**) and twenty-five OA patients’ (**1**–**25**) synovial fluids (10%) using the nLuc-hPAR1/2/4-eYFP-CHO reporter cell lines (Fig. 2). A significantly higher level of PAR1 cleavage was detected in OA patient synovial fluids treated cells compared to the healthy fluid treated cells (Fig. 2A). Interestingly, cleavage of PAR2 was not significantly different between healthy and OA synovial fluids (Fig. 2B), while cleavage of PAR4 was significantly lower (Fig. 2C) with OA patient knee joint fluids compared to the healthy control samples. It must be noted that these assays were done with a 1:10 dilution of the knee joint fluids and indicated cleavage of ~ 10–20% of maximum thrombin (3 U ml^−1^) activity in PAR1 and ~ 15–20% of maximum trypsin (100 nM) activity in PAR2, respectively. This would translate to the presence of ~ 3–6 U ml^−1^ of thrombin-like enzymatic activity and 15–20 nM trypsin-like enzymatic activity in the knee joint fluids. Sample sizes were not large enough in this study to correlate enzyme activity levels with radiological OA severity.

To examine if PAR cleavage activity in synovial fluids is influenced by patient characteristics, we stratified the PAR1, PAR2, and PAR4 cleavage data further based on patient demographics including sex, BMI, and age (Figure S1). Interestingly, PAR1 (Figure S1-A(i)) and PAR2 (Figure S1-B(i)) cleavage was higher with synovial fluids from males compared to females, while PAR4 cleavage was higher in females relative to males (Figure S1-C(i)). There were no differences seen in PAR1, PAR2, or PAR4 cleavage activity between synovial fluids from normal weight (BMI 18.5–24.9 kg m^−2^), overweight (BMI 25.0–29.9 kg m^−2^), and obese (BMI > 29.9 kg m^−2^) patients (Figure S1-(ii)) as well as across different age groups (Figure S1-(iii)). Given the relatively small sample size in different categories, the trends observed here are interesting but need to be confirmed in a larger patient cohort in future studies.

### Classification of PAR cleaving enzymes in synovial fluids

In order to understand the class/type of PAR cleaving enzymes present in the synovial fluids, we used enzyme inhibitors targeting serine proteinases that are known to activate PARs. Thrombin-like enzymes were inhibited with PPACK, a potent, irreversible, thrombin selective inhibitor^61,62^, which can also inhibit coagulation factors VIIa and XIIa, tissue plasminogen activator (tPA), kallikrein^63^, and urokinase^64^. Trypsin-like enzymes were inhibited with STI, an inhibitor of trypsin^65^ that can also inhibit chymotrypsin^65^, matriptase^66^, plasmin, and plasma kallikrein^67^. Metalloproteinases were inhibited with BB-94 and ONO-4817. BB94 is a broad-spectrum metalloprotease inhibitor targeting peptidases in families M10 and M12, including MMP-1, 2, 3, 7, 8, 9, 12, 13, 28, ADAM-8, 19, DEC1, and meprin-α, β^68–70^, whereas MMP inhibitor ONO-4817 targets MMP-2, 3, 7, 8, 9, 12, 13^71–74^.

**Figure 3:**
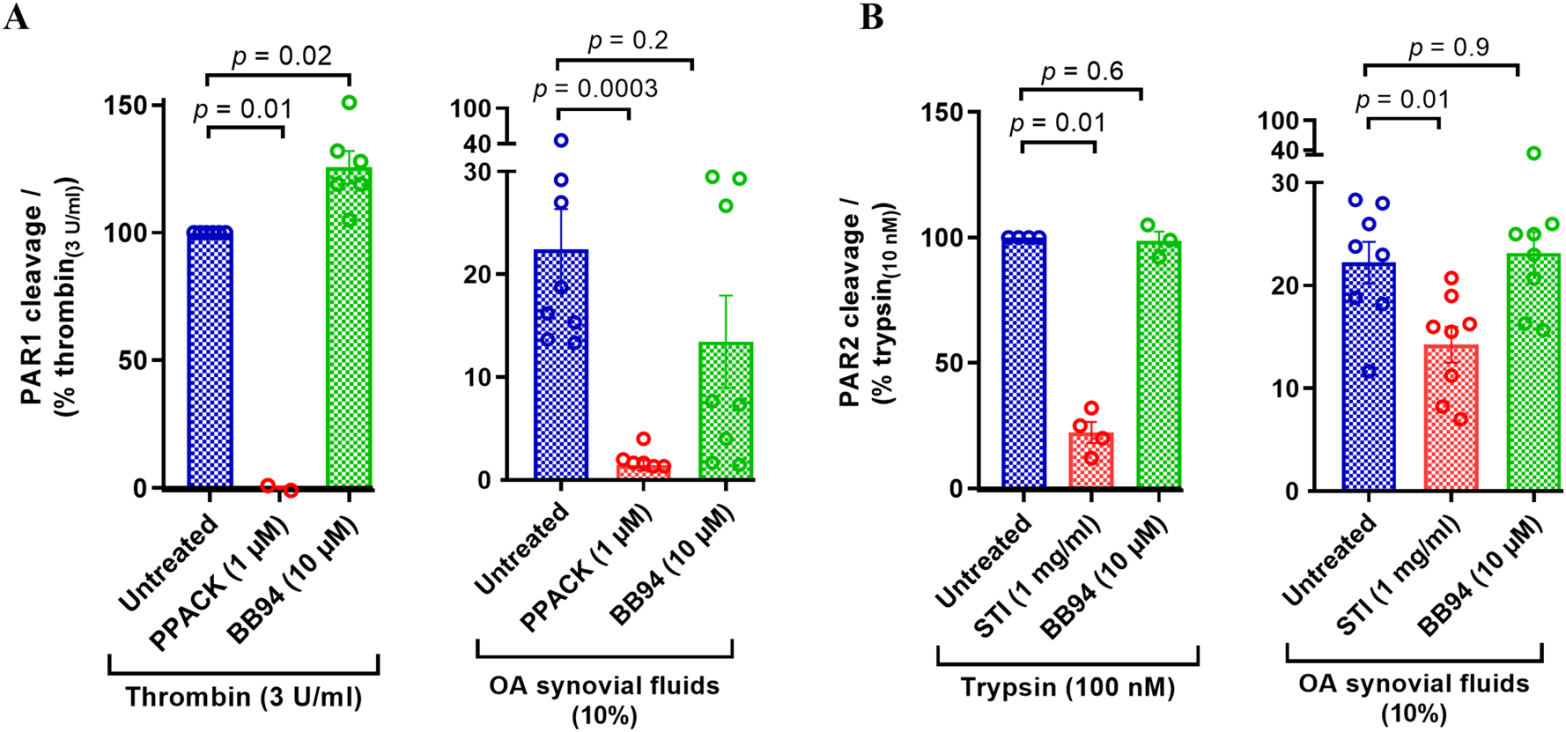
Effect of protease inhibitors on PAR cleavage by eight human OA knee joint synovial fluids. Standard PAR activating enzymes (thrombin for PAR1 and trypsin for PAR2) or synovial fluids (10%) were pretreated with an enzyme-selective inhibitor, PPACK, STI or BB-94, before addition to nLuc-hPAR-eYFP-CHO cells. Release of the N-terminal nLuc tag into the culture supernatants was monitored as an index of receptor N-terminus cleavage. (**A**) PAR1 cleavage and (**B**) PAR2 cleavage. Each bar represents the mean ± SEM (*N* = 3–6). One-Way ANOVA Kruskal–Wallis test was utilized to assess differences between groups. *p* < 0.05 was considered statistically significant.

PAR1 and PAR2 cleavage by a subset of synovial fluid samples (1, 3, 5, 10, 17, 19, 20, 23) pretreated with various enzyme inhibitors was examined using the reporter cell lines (Fig. 3). Since we saw a decrease in PAR4 cleavage by OA patient knee joint fluids (Fig. 2C) we did not examine cleavage of this receptor with inhibitor treated synovial fluids. The limited volume of synovial fluids available from healthy volunteers prevented inclusion of these samples. As expected, cleavage of PAR1 by thrombin (3 U ml^−1^) was completely blocked by PPACK (1 µM, Fig. 3A) and the PAR2 cleavage by trypsin (10 nM) was significantly blocked by STI (1 mg ml^−1^, Fig. 3B), and was not blocked by BB-94 (10 µM, Fig. 3). The patient synovial fluids (10%) pretreated with PPACK showed almost a complete reduction in PAR1 cleavage, whereas BB-94 treated fluids did not show a significant drop in PAR1 cleavage (Fig. 3A). Similarly, STI treated fluids showed a significant drop in PAR2 cleavage and BB-94 treatment of synovial fluids did not have an effect on PAR2 cleavage (Fig. 3B). Overall, the incomplete inhibition of PAR1 and PAR2 cleavage by any single class of proteinase inhibitor indicates that OA patient synovial fluids contain multiple serine proteinases and metalloproteinases that cleave PARs expressed in the knee joint.

**Figure 4:**
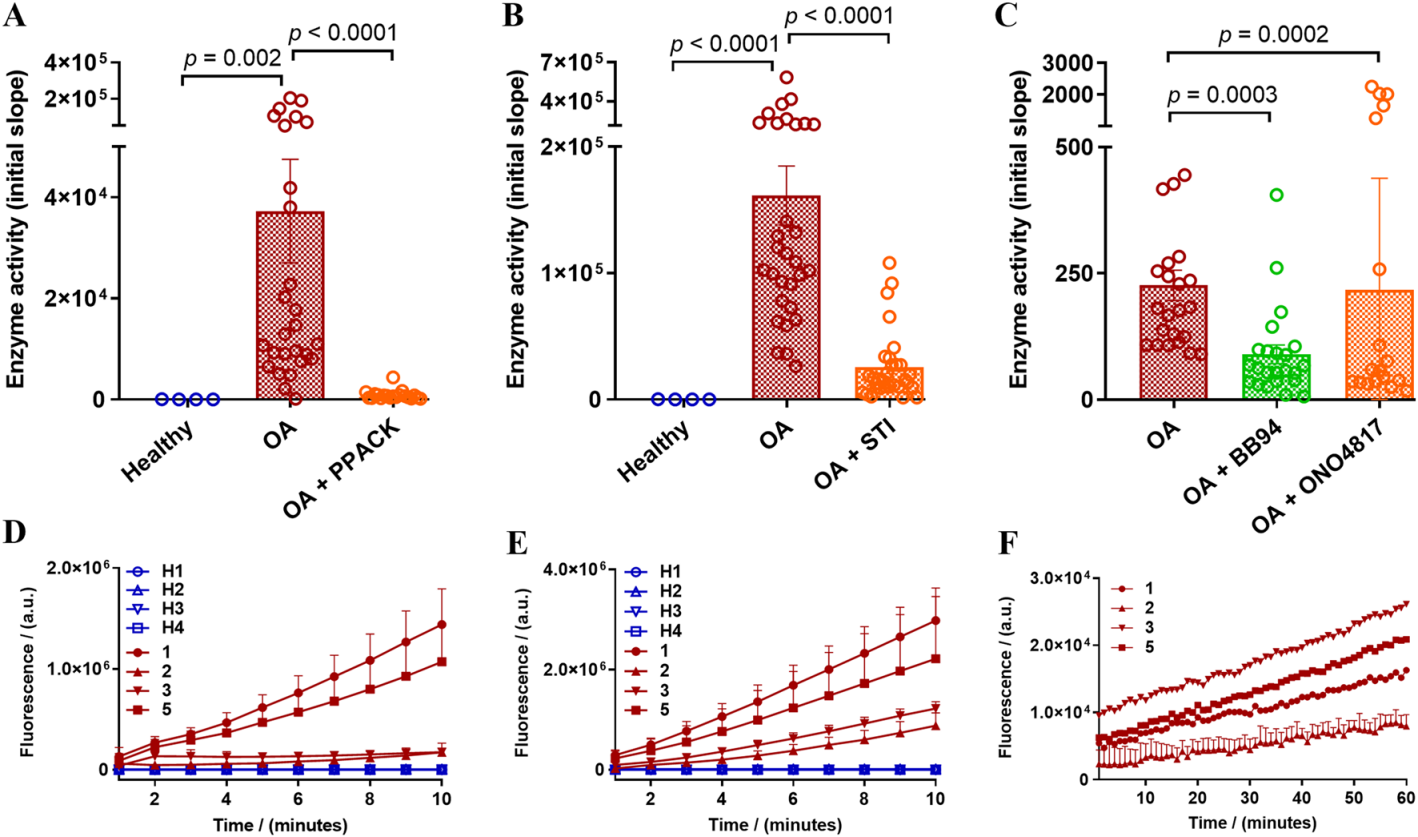
Cleavage of enzyme class preferred fluorogenic substrates by healthy and OA knee joint synovial fluids. Fluorogenic substrates were incubated with the healthy and OA synovial fluids (10%) with and without enzyme inhibitors (PPACK, STI, BB-94 or ONO-4817) and cleavage of (**A**) Bz-FVR-AMC (thrombin-like enzymes), (**B**) Boc-QAR-AMC (trypsin-like enzymes), and (**C**) MCA-KPLGL-Dpa(DNP)-AR-NH_2_ (MMPs) substrates was monitored. Representative kinetic traces of four healthy and four patient samples obtained with (**D**) Bz-FVR-AMC (10 min) and (**E**) Boc-QAR-AMC (10 min), and (**F**) four patient samples with MCA-KPLGL-Dpa(DNP)-AR-NH_2_ (60 min) substrates. The data represents the mean ± SEM (*N* = 1–3). One-Way ANOVA Kruskal–Wallis test was utilized to assess differences between groups. *p* < 0.05 was considered statistically significant.

To further clarify the nature of different enzymes present in the synovial fluids, we used peptide-based fluorogenic substrates of thrombin, trypsin, chymotrypsin, urokinase, and MMPs. With the use of thrombin substrate Bz-Phe-Val-Arg-AMC (Figure S2-A), trypsin and matriptase substrate Boc-Gln-Ala-Arg-AMC (Figure S2-B), and a broad-spectrum MMP substrate MCA-Lys-Pro-Leu-Gly-Leu-Dpa(DNP)-Ala-Arg-NH_2_ which can be hydrolyzed by MMP-1, 2, 3, 7, 8, 9, 12, 13, 14, 16, 20, ADAM-10, 17/TACE and BACE2^75–78^, significant levels of thrombin-like enzymes (Fig. 4A), trypsin-like enzymes (Fig. 4B) and MMPs (Fig. 4C) activity were detected in the twenty-five patients’ synovial fluids (10%) tested. However, no cleavage of the chymotrypsin sensitive substrate Suc-Ala-Ala-Pro-Phe-AMC, or the urokinase and plasminogen activators sensitive substrates Z-Gly-Gly-Arg-AMC.HCl was detected with any of the twenty-five patient synovial fluids (data not shown). Remarkably, thrombin-like and trypsin-like enzymes were higher in OA samples compared to healthy synovial fluids (Fig. 4A, B), which was consistent with the high PAR1 cleavage found with the OA patient samples in the PAR biosensor assay (Fig. 2A). The limited volume of synovial fluids available from healthy volunteers prevented assessment of healthy samples with the MMP substrate.

As observed in the PAR biosensor cleavage assay (Fig. 3), significant inhibition of thrombin-like (Fig. 4A), trypsin-like (Fig. 4B) and MMP (Fig. 4C) enzymes activity was found in all the OA patient synovial fluids pretreated with PPACK (1 µM), STI (1 mg ml^−1^) or BB-94 (10 µM), respectively. In addition, here we used another MMP inhibitor ONO-4817 and as observed with BB-94, a significant reduction in MMP activity was seen in the ONO-4817 (10 µM) pretreated patients’ synovial fluids (Fig. 4C). This suggests that one or more MMP is present in OA synovial fluids. Representative enzymatic activity kinetic curves are shown in Fig. 4D–F. Together, this set of experiments further confirmed the presence of significant levels of serine proteinases and matrix metalloproteinases in the OA knee joint fluids.

The substrate cleavage enzyme activity data was also analyzed in the context of the OA patient demographics (Figure S3). However, there was no substantial difference seen in enzyme activity by sex of the patients. This contrasts with the observations seen with the PAR biosensor cleavage assay data (Figure S1-(i)). As seen with PAR cleavage, enzyme activity did not show a significant difference between normal weight, overweight, or obese patients. It was however interesting to see a significantly lower level of thrombin-like enzyme activity in older adults relative to middle-aged adults (Figure S3-A(iii)). There was no significant difference seen in the trypsin-like enzyme activity between different age groups (Figure S3-B(iii)) in agreement with observations with the PAR2 cleavage data (Figure S1-B(iii)). Interestingly, MMP activity was significantly higher in older adults compared to young or middle-aged patients (Figure S3-C(iii)). Together, there seems to be an interesting link between OA patient demographics, enzyme activity, and PAR cleavage. The sample size in this human pilot study was however not large enough to make a strong statistical comparison and these observations needs to be confirmed in a larger patient cohort in the future.

### OA joint fluid activation of PAR mediated calcium signaling

**Figure 5:**
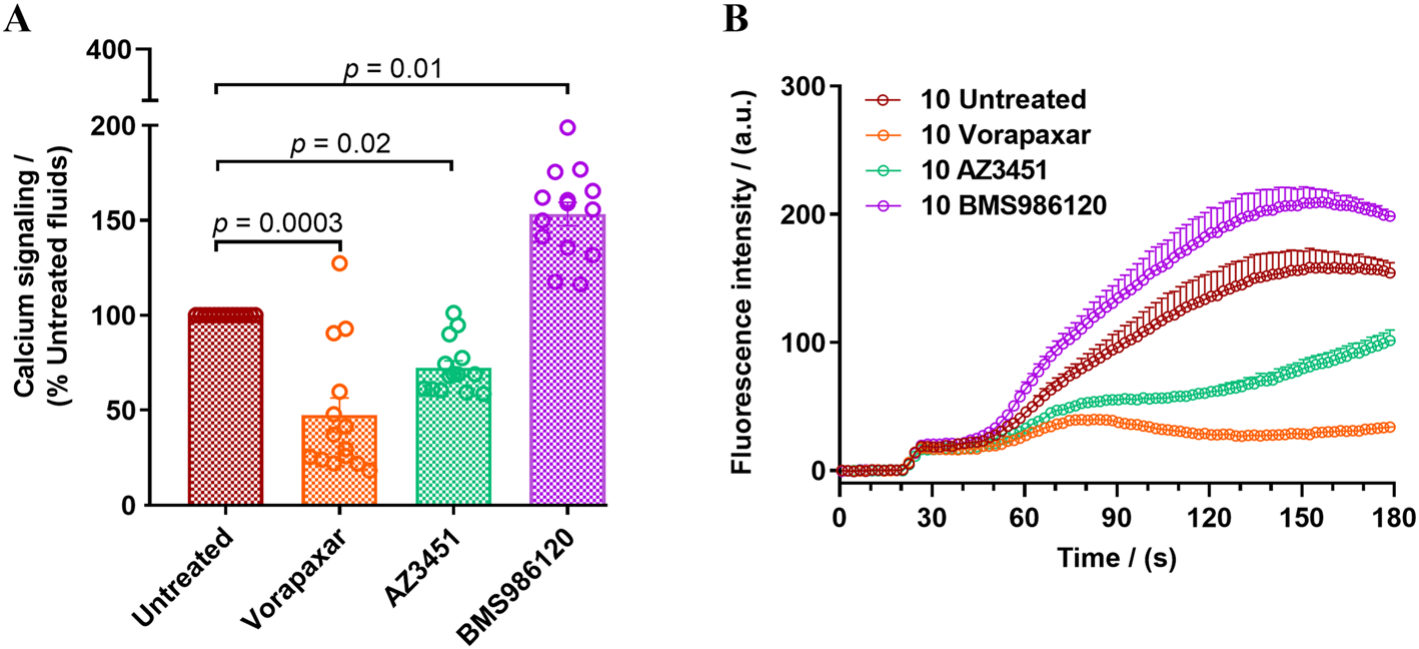
OA joint fluid activation of PAR mediated calcium signaling. Synovial fluids (10%) were added to HEK293 cells expressing PAR1, PAR2 and PAR4. Elevation in intracellular calcium levels were monitored in (**A**) untreated cells and cells pretreated with PAR antagonists, Vorapaxar, 1000 nM (PAR1 antagonist), AZ3451, 1000 nM (PAR2 antagonist) and BMS986120, 1000 nM (PAR4 antagonist). Scatter plots depict peak calcium signaling levels in individual experiments as a percentage of the response elicited by the synovial fluid samples in untreated cells. One-Way ANOVA Kruskal–Wallis test was utilized to assess differences between groups. *p* < 0.05 was considered statistically significant. (**B**) A representative calcium signaling trace obtained for PAR mediated calcium signaling triggered by one patient sample in the absence and presence of PAR1, PAR2, and PAR4 antagonists. The data represent the mean ± SEM of three independent experiments (*N* = 3) performed in duplicate.

In order to determine if PAR cleaving proteinases in the synovial fluids activate PAR signaling downstream, fourteen OA samples (1–3, 5, 10, 12, 15–17, 19, 22–25) were characterized through calcium signaling assay in PAR1, PAR2 and PAR4 expressing HEK293 cells. It is well established that PAR activating enzymes, thrombin (PAR1 and PAR4) and trypsin (PAR2), and PAR specific agonist peptides, TFLLR-NH_2_ (PAR1), SLIGRL-NH_2_ (PAR2) and AYPGKF-NH_2_ (PAR4) trigger robust calcium release (Figure S4-A) that is dependent on Gα_q/11_-coupled signaling pathway^52,79–81^. PAR mediated calcium signaling can be inhibited using the antagonists, Vorapaxar (PAR1), AZ3451 (PAR2) and BMS-986120 (PAR4)^79^, in a concentration-dependent manner (Figure S4-B). OA synovial fluids (10%) triggered an increase in calcium signaling in HEK293 cells expressing PAR1, PAR2 and PAR4 (Figs. 5 and S5). Pre-treatment of cells with Vorapaxar (PAR1) and AZ3451 (PAR2) resulted in a significant drop in calcium signaling (Figs. 5 and S5) consistent with our PAR cleavage data where PAR1 and PAR2 cleavage was monitored (Fig. 2A, B). Curiously, a significant increase in intracellular calcium was seen in cells that had been treated with the PAR4 antagonist BMS-986120 prior to OA synovial fluid treatment (Figs. 5 and S5-B).

### Correlation of KOOS, WOMAC, and VAS scores to PAR1/2/4 activity

OA patients self-reported arthritic questionnaires scores including Knee Injury and Osteoarthritis Outcome Score (KOOS)^48^, the Western Ontario and McMaster Universities Arthritis Index (WOMAC)^49^ and Visual Analog Scale (VAS) pain score^50^ at baseline prior to surgery (Table S1) were correlated to the obtained PAR1, PAR2, and PAR4 cleavage data to identify if any correlation exists (Figure S6). Based on the Pearson correlation coefficient interpretation scale described under Materials and Methods section, a weak positive correlation was observed between PAR1 cleavage and KOOS [*r*_Pain_ = 0.30, *r*_Symptom_ = 0.17, *r*_ADL_ = 0.17, *r*_Sport/Rec_ = 0.24, *r*_QOL_ = 0.34 (Figure S6-A)], WOMAC [*r*_Pain_ = 0.28, *r*_Physical function_ = 0.17, *r*_Stiffness_ = 0.14 (Figure S6-B)], and VAS [*r*_Move_ = 0.25 (Figure S6-C)] scores. At rest, the VAS score had a weak negative correlation (*r*_Rest_ = -0.15) against PAR1 activity. However, against the PAR2 activity, there was a weak positive correlation found only with one subcategory of the KOOS (*r*_QOL_ = 0.11), WOMAC (*r*_Pain_ = 0.13), and VAS (*r*_Move_ = 0.24) score, and a weak negative correlation was found with one of the KOOS (*r*_Symptom_ =  − 0.21) and WOMAC (*r*_Stiffness_ =  − 0.15) score, while all other scores had negligible correlation (Figure S6). As observed with the PAR1 correlation, most of the KOOS (*r*_Pain_ = 0.17, *r*_ADL_ = 0.20) and WOMAC (*r*_Pain_ = 0.22, *r*_Physical function_ = 0.20) scores had a weak positive correlation against the PAR4 activity, although two of the KOOS (*r*_Sport/Rec_ = − 0.10, *r*_QOL_ =  − 0.18) had a weak negative correlation, and other subcategories and VAS scores had negligible correlation (Figure S6). Overall, the sample sizes in the current study are not sufficient to do robust correlation analysis however such analysis of larger patient cohorts is likely to be informative.

## Discussion

Using novel biosensor expressing reporter cells and fluorogenic substrates we have monitored PAR cleavage and proteolytic activity in knee joint fluids from twenty-five OA patients with disease severity ranging from KL grade 1–3. We find multiple active PAR1, PAR2, and PAR4 activating enzymes, including serine proteinases and metalloproteinases, in human knee joint synovial fluids tested. Curiously, levels of PAR1 activating enzymes were elevated and PAR4 activating enzyme levels decreased in the OA joint fluids but not in the healthy joint fluids studied here. This decline in PAR4 activating enzymes in OA joint fluids is interesting since some PAR1 and PAR2 activating enzymes, including thrombin and trypsin, also activate PAR4. This divergent activation of PAR1 and PAR4 in OA suggests that there are distinct PAR1 and PAR4 activating enzymes in the knee joint that should be identified in order to better understand the roles of these receptors in OA pathogenesis.

Proteolytic enzymes are important mediators of joint health and pathophysiology. The role of MMPs in particular, as a class of enzymes that digest ECM components and cause cartilage degradation, is well established^82^. More recently it has emerged that other classes of enzymes such as serine proteinases are also upregulated in OA and may participate in proteolytic cascades leading to cartilage destruction^83^. Many of the enzymes that are implicated in OA pathology also trigger pro-inflammatory signaling cascades through the activation of the PAR family of GPCRs^8^.

PARs have been studied in the context of arthritis using several animal models. PAR1 deficient mice showed decreased cartilage degradation, and lower levels of synovial cytokine mRNA and MMP-13 mRNA in a model of antigen-induced arthritis^28^. PAR1 deficiency is also protective in a model of psoriatic arthritis driven by increased dermal expression of kallikrein 6^84^. In contrast, PAR1 deficiency did not afford significant protection in the destabilization of the medial meniscus (DMM) model of OA^27^. PAR2 deletion or pharmacological inhibition on the other hand has shown more consistent protective effects across multiple models, though there are some variations in the degree of reduction in cartilage erosion and subchondral bone thickening reported^14,17,27^. The role of PAR4 expressed in joint cells or tissues is not as well studied, however PAR4 activation significantly inhibits PAR2 agonist and transient receptor potential vanilloid-4 (TRPV4) agonist-mediated visceral pain^85^ and PAR4 activation can also decrease excitability in dorsal root ganglion neurons^86^. PAR4 signaling may therefore be protective in the context of osteoarthritis pain. Overall, the disparate results across different models point to PAR signaling in different immune and joint cells contributing to the disease, with PAR expression well established in chondrocytes, fibroblast-like synoviocytes, osteoblasts, immune cells, and nociceptors^8^. Further studies with tissue-specific deletion of PARs across multiple models of arthritis as well as careful analysis of possible species differences are required to fully understand the relative contributions of each receptor and cell type in human OA.

Several proteolytic enzyme activators of PARs are nonetheless implicated in OA and similar protection of joints in OA is also reported when the key PAR1 and PAR2 activating enzymes are inhibited. Serine proteinases involved in the coagulation and fibrinolysis cascades, including thrombin, plasminogen activators and plasmin, are well established as activators of PAR1 and show a substantial increase in the inflamed joint of OA patients and animal models^87,88^. MMPs including MMP-1, 2, 3, 9, 13 and 14 are secreted in response to inflammatory cytokines and growth factors by chondrocytes and synoviocytes^4,82^. A number of these MMPs including MMP-1, 2, 3, 8, 9, 12 and 13 are able to activate PARs. In addition, immune cell-derived proteinases such as mast cell tryptase, neutrophil elastase, proteinase-3 and cathepsins are also able to cleave and activate PAR1 and PAR2^9,24^. In this regard, our finding that multiple OA joint fluid enzymes can cleave PARs is particularly relevant. Firstly, this suggests that specific inhibition of individual enzymes may not be useful in the treatment of OA since other enzymes could still perpetuate inflammatory signaling through these receptors. Pharmacological inhibition of individual receptors may instead be more beneficial. Secondly, in recent years it has emerged that not all enzymes trigger identical signaling responses through a PAR receptor, a concept called biased signaling^9,42^ that is now widely seen across multiple GPCRs. In addition to the canonical activation site of PARs [thrombin activation of PAR1 (cleavage at Arg^41^/Ser^42^) or PAR4 (cleavage at Arg^47^/Gly^48^) and trypsin activation of PAR2 (cleavage at Arg^36^/Ser^37^)], PAR cleavage by other enzymes occurs at distinct sites on the receptor N-terminus to reveal novel tethered-ligands and activate biased signaling responses^24^. For example, cleavage of PAR1 by MMP (Asp^39^/Pro^40^, Leu^44^/Leu^45^, Phe^87^/Ile^88^), neutrophil elastase (Ala^36^/Thr^37^, Val^72^/Ser^73^, Arg^86^/Phe^87^) and proteinase-3 (Aal^36^/Thr^37^, Pro^48^/Asn^49^, Val^72^/Ser^73^, Ala^92^/Ser^93^) is reported to occur at multiple sites that differ from the thrombin cleavage site^24,80^. In the case of PAR2, neutrophil elastase (Ala^66^/Ser^67^, Ser^67^/Val^68^)^38^ and cathepsin-S (Gly^40^/Lys^41^)^39^ also cleave the receptor at different sites than trypsin. Each of these different cleavage events can result in different signaling responses, some of which may be protective. While we show that PARs can be cleaved by various enzymes in OA joint fluids, a thorough characterization of receptor coupling to different G protein and β-arrestin mediated signaling pathways is necessary to fully understand the role of this signaling system in joint health and pathology. The diversity of PAR activators in the joints also highlight the importance of understanding signaling differences elicited by the different PAR activating enzymes in testing appropriate therapeutic interventions.

Interestingly, the significant reduction in OA joint fluid triggered calcium signaling in the presence of a PAR1 antagonist along with the elevation of PAR1 cleaving enzyme activity in OA synovial fluids suggests that inhibition of PAR1 could be a promising therapeutic strategy to explore in OA. On the other hand, inhibition of PAR4 led to an increase in calcium signaling indicating that PAR4 mediated signaling may be producing factors or signaling events that inhibit responses from PAR1 and PAR2. Given the significant decrease in PAR4 activating enzyme levels in OA patients, this suggests the intriguing hypothesis that PAR4 signaling is a protective response in the knee joint that is lost in OA. While a significant difference between healthy and OA synovial fluid PAR2 activating enzymes was not noted in our study, PAR2 activating enzymes are nevertheless present in the knee joint synovial fluid. PAR2 is known to be an important regulator of immune cell infiltration and may contribute to OA pathogenesis. Further analysis of samples from different types and severity of OA are required to fully correlate PAR activating protease levels with disease pathogenesis.

In summary, we have established and characterized here a novel biosensor expressing cell line and an assay that allows rapid and facile screening of PAR cleaving enzymes in complex biological fluids. In a small population of OA patients, we were able to detect multiple enzymes that target PARs and find elevated levels of PAR1 cleaving enzymes in OA. We further verified that PAR cleavage led to increases in receptor mediated calcium signaling that could be inhibited using PAR1 and PAR2 specific antagonists. This study broadly defines proteinase classes that are present in the arthritic joint and guide future studies aimed at isolating and further characterizing endogenous regulators of PAR receptors in the healthy and diseased joints.

## Supplementary Information

Supplementary Information.

## Data Availability

All data generated or analysed during this study are included in this published article and its Supplementary Information file.

## Abbreviations

OA: Osteoarthritis
PARs: Proteinase-activated receptors
GPCR: G protein-coupled receptor
ECM: Extracellular matrix
MMPs: Matrix metalloproteinases
