# Supplementary material for: Human osteoarthritis knee joint synovial fluids cleave and activate Proteinase-Activated Receptor (PAR) mediated signaling
Source: Sci Rep. 2023 Jan 20;13:1124. doi: 10.1038/s41598-023-28068-3 (PMC9859807; doi:10.1038/s41598-023-28068-3)
Supplement: Supplementary file 1 — Supplementary Information. [file 41598_2023_28068_MOESM1_ESM.docx]

**Human osteoarthritis knee joint synovial fluids cleave and activate Proteinase-Activated Receptor (PAR) mediated signaling.**

Arundhasa Chandrabalan^a^, Andrew Firth^b^, Robert B Litchfield^b^, C Thomas Appleton^a,c^, Alan Getgood^b^ and Rithwik Ramachandran^a*^

^a^ *Department of Physiology and Pharmacology, Bone and Joint Institute, Schulich School of Medicine and Dentistry, University of Western Ontario, London, Ontario, Canada.*

^b^ *Division of Orthopedic Surgery, Bone and Joint Institute, Fowler Kennedy Sport Medicine Clinic, Schulich School of Medicine and Dentistry, University of Western Ontario, London, Ontario, Canada.*

^c^ *Department of Medicine,* *Bone and Joint Institute, The Dr. Sandy Kirkley Centre for Musculoskeletal Research, Schulich School of Medicine and Dentistry, London, Ontario, Canada.*

*Correspondence and material requests to:

Dr. Rithwik Ramachandran, Department of Physiology and Pharmacology, University of Western Ontario, London, ON, N6A 5C1. [rramach@uwo.ca](mailto:rramach@uwo.ca), Tel: 519-661-2142

**SUPPLEMENTARY DATA**

**Table S1.** KOOS, WOMAC and VAS scores of the OA patients. KOOS, Knee Injury and Osteoarthritis Outcome Score; WOMAC, Western Ontario and McMaster Universities Osteoarthritis Index; VAS, Visual Analog Scale; ADL, Activities in Daily Living function; Sport/Rec, Sport and Recreation function; QOL, Quality of Life

| Patient | KOOS / (%) | | | | | | WOMAC / (%) | | | | VAS / (cm) | |
| --- | --- | --- | --- | --- | --- | --- | --- | --- | --- | --- | --- | --- |
|  | Pain | Symptom | ADL | Sport/Rec | QOL | Total | Pain | Stiffness | Physical function | Total | Rest | Move |
| 1 | 83 | 71 | 90 | 70 | 63 | 75 | 90 | 88 | 90 | 90 | 0.5 | 5.9 |
| 2 | 44 | 57 | 53 | 0 | 31 | 37 | 53 | 50 | 38 | 51 | 8.2 | 9.4 |
| 3 | 56 | 61 | 60 | 45 | 44 | 53 | 65 | 50 | 60 | 60 | 5.5 | 7.7 |
| 4 | 64 | 61 | 76 | 30 | 19 | 50 | 80 | 75 | 76 | 77 | 0.0 | 7.7 |
| 5 | 36 | 61 | 62 | 0 | 6 | 33 | 25 | 38 | 62 | 52 | 9.8 | 6.3 |
| 6 | 72 | 75 | 88 | 60 | 31 | 65 | 88 | 80 | 88 | 86 | 0.7 | 2.5 |
| 7 | 56 | 36 | 76 | 30 | 13 | 42 | 65 | 38 | 76 | 71 | 2.6 | 5.7 |
| 8 | 75 | 46 | 87 | 25 | 31 | 53 | 85 | 50 | 87 | 83 | 0.9 | 3.8 |
| 9 | 83 | 64 | 93 | 20 | 6 | 53 | 93 | 100 | 75 | 93 | 0.8 | 1.5 |
| 10 | 31 | 29 | 29 | 0 | 31 | 24 | 40 | 13 | 29 | 30 | 9.4 | 9.7 |
| 11 | 47 | 43 | 54 | 10 | 6 | 32 | 54 | 60 | 50 | 55 | 0.0 | 7.8 |
| 12 | 44 | 39 | 43 | 0 | 38 | 33 | 50 | 38 | 43 | 44 | 5.5 | 5.8 |
| 13 | 50 | 25 | 65 | 0 | 13 | 30 | 70 | 13 | 65 | 61 | 0.0 | 6.3 |
| 14 | 64 | 68 | 62 | 0 | 6 | 40 | 62 | 70 | 63 | 64 | 5.0 | 7.9 |
| 15 | 53 | 50 | 75 | 30 | 31 | 48 | 60 | 38 | 75 | 69 | 1.1 | 6.6 |
| 16 | 19 | 25 | 21 | 0 | 0 | 13 | 10 | 13 | 21 | 18 | 3.8 | 3.5 |
| 17 | 47 | 43 | 46 | 0 | 13 | 30 | 55 | 50 | 46 | 48 | 1.6 | 3.6 |
| 18 | 56 | 57 | 71 | 40 | 25 | 50 | 65 | 75 | 71 | 70 | 1.9 | 2.9 |
| 19 | 44 | 36 | 60 | 0 | 6 | 29 | 50 | 25 | 60 | 55 | 9.1 | 7.3 |
| 20 | 39 | 57 | 53 | 15 | 13 | 35 | 45 | 38 | 53 | 50 | 2.2 | 7.6 |
| 21 | 58 | 61 | 63 | 25 | 25 | 46 | 63 | 70 | 50 | 64 | 5.0 | 5.3 |
| 22 | 47 | 57 | 66 | 20 | 38 | 46 | 50 | 75 | 66 | 64 | 1.0 | 2.8 |
| 23 | 36 | 18 | 28 | 5 | 38 | 25 | 45 | 25 | 28 | 31 | 4.8 | 8.0 |
| 24 | 44 | 39 | 60 | 5 | 6 | 31 | 65 | 63 | 60 | 61 | 5.3 | 5.3 |
| 25 | 25 | 29 | 29 | 5 | 19 | 21 | 29 | 25 | 13 | 27 | 6.9 | 8.2 |

**
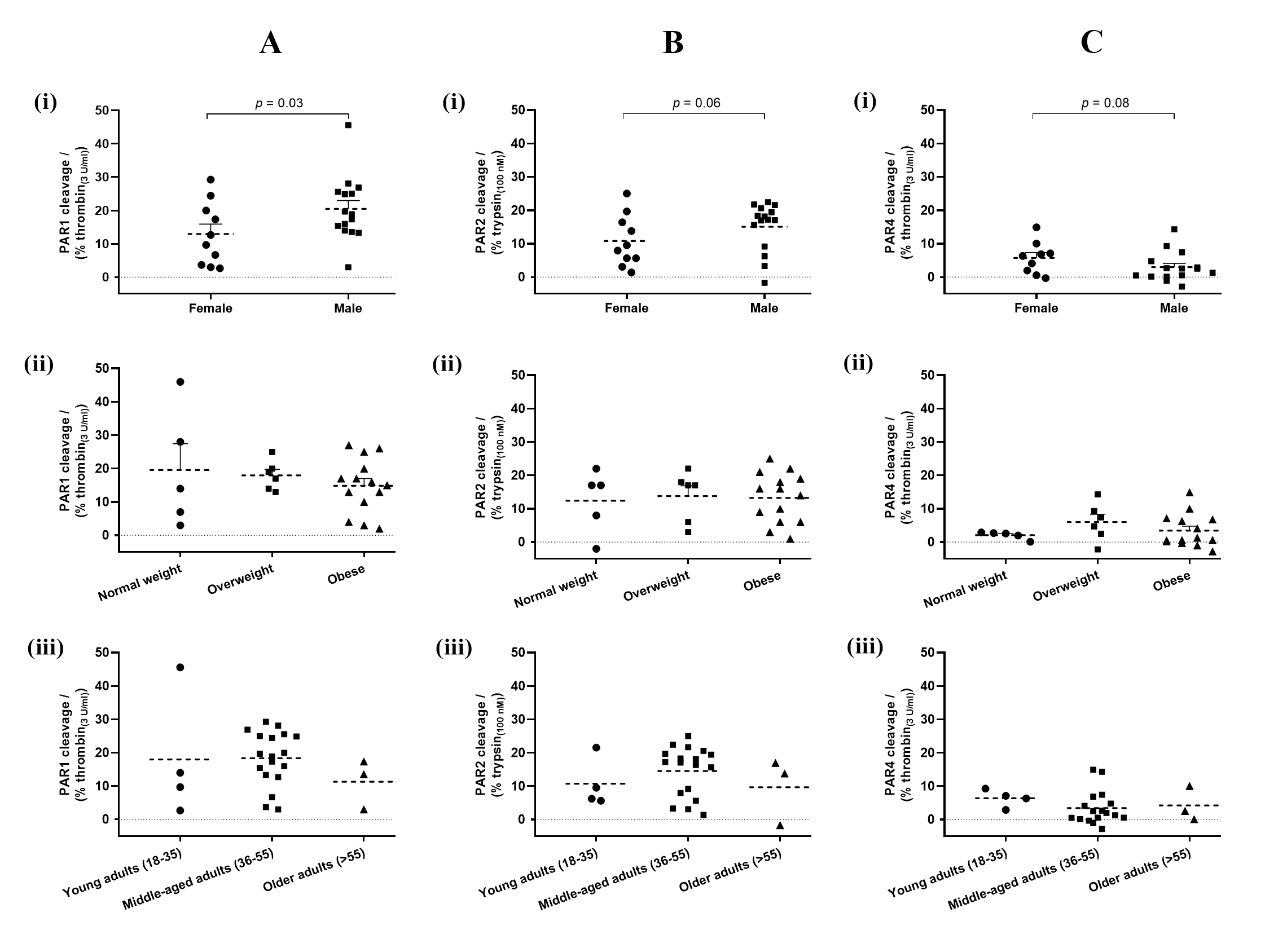
**

**Figure S1.** Cleavage of PAR1 (panel **A**), PAR2 (panel **B**) and PAR4 (panel **C**) by OA patient synovial fluids were plotted as a function of patient demographics, **(i)** sex, **(ii)** BMI and **(iii)** age. Each data point on the scatter dot plot represents the mean ± SEM (*N* ≥ 3). Mann-Whitney U test was utilized to assess any statistical significance between two groups, and Kruskal-Wallis test was utilized to compare more than two groups. *p* < 0.05 was considered to be statistically significant.

**
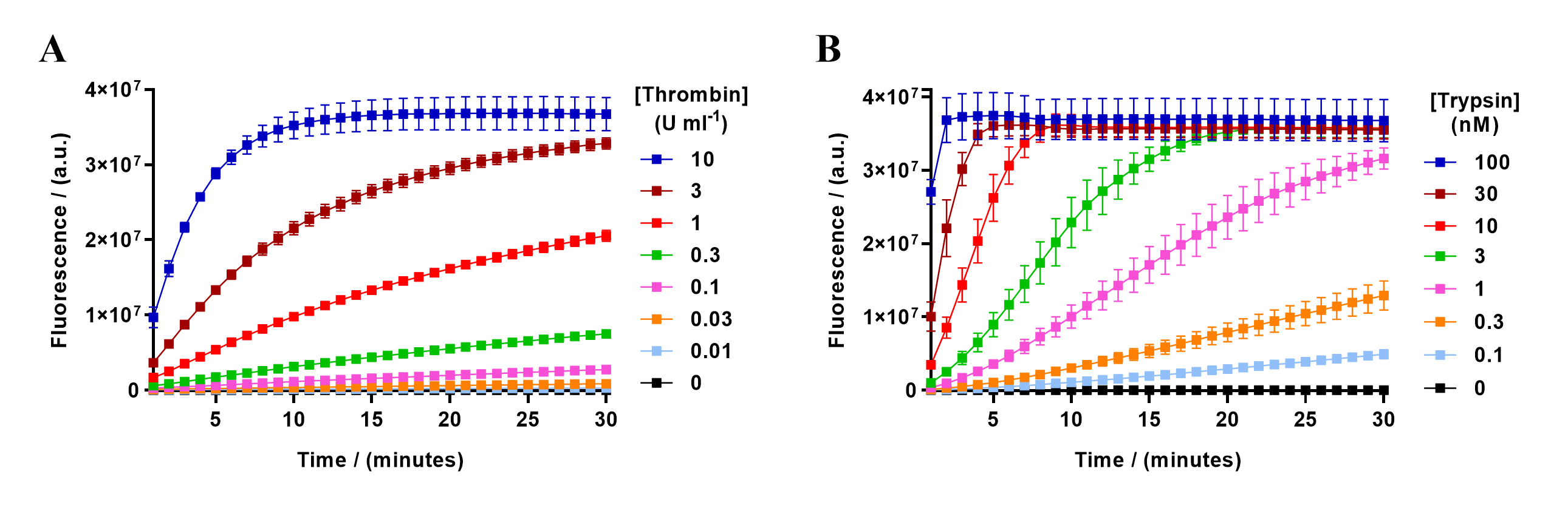
**

**Figure S2:** Kinetic curves of the thrombin and trypsin fluorogenic peptide substrate hydrolysis, (**A**) Bz-FVR-AMC (100 µM) cleavage by thrombin (0.03 - 10 U ml^-1^), and (**B**) Boc-QAR-AMC (100 µM) cleavage by trypsin (0.1 - 100 nM). Each data point on the curve represents the mean ± SEM (*N* = 3).


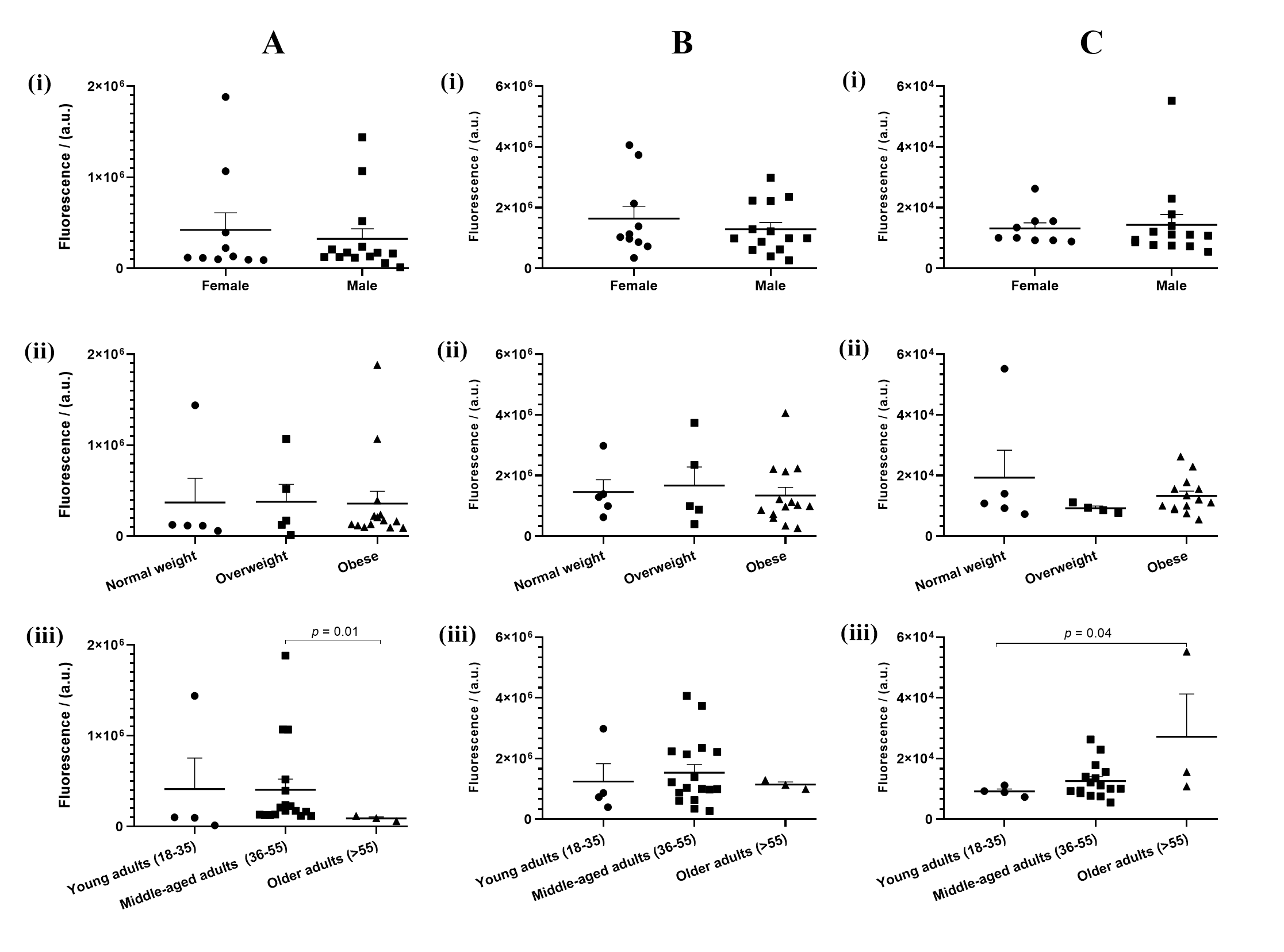


**Figure S3.** Cleavage of fluorogenic substrates Bz-FVR-AMC (thrombin-like enzymes, panel **A**), Boc-QAR-AMC (trypsin-like enzymes, panel **B**) and MCA-KPLGL-Dpa(DNP)-AR-NH_2_ (MMPs, panel **C**) by enzymes in OA patient synovial fluids plotted as a function of patient demographics, **(i)** sex, **(ii)** BMI and **(iii)** age. Each data point on the scatter dot plot represents the mean ± SEM (*N* ≥ 3). Mann-Whitney U test was utilized to assess differences between two groups, and Kruskal-Wallis test was utilized to compare more than two groups. *p* < 0.05 was considered to be statistically significant.

**
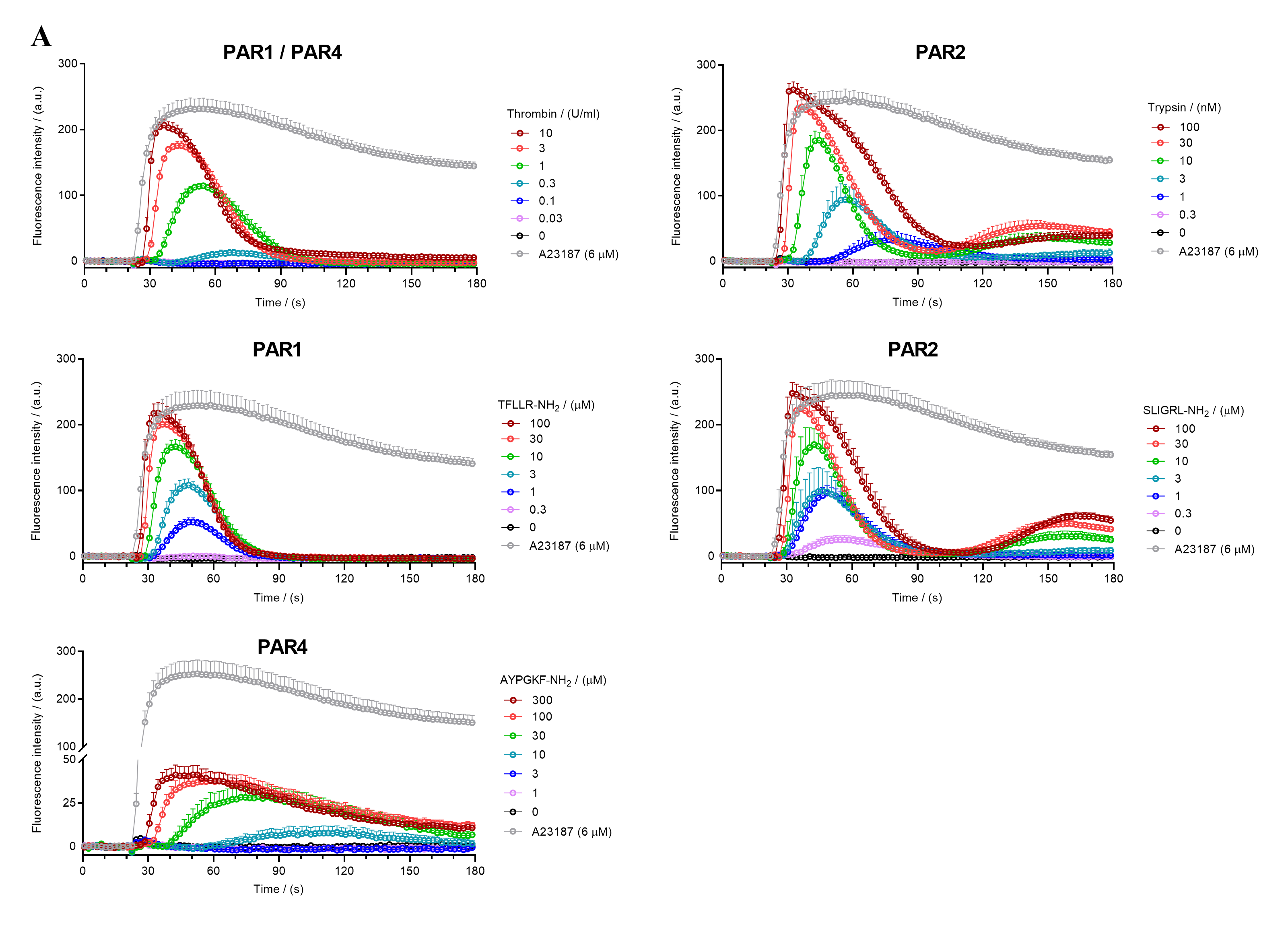
**

**
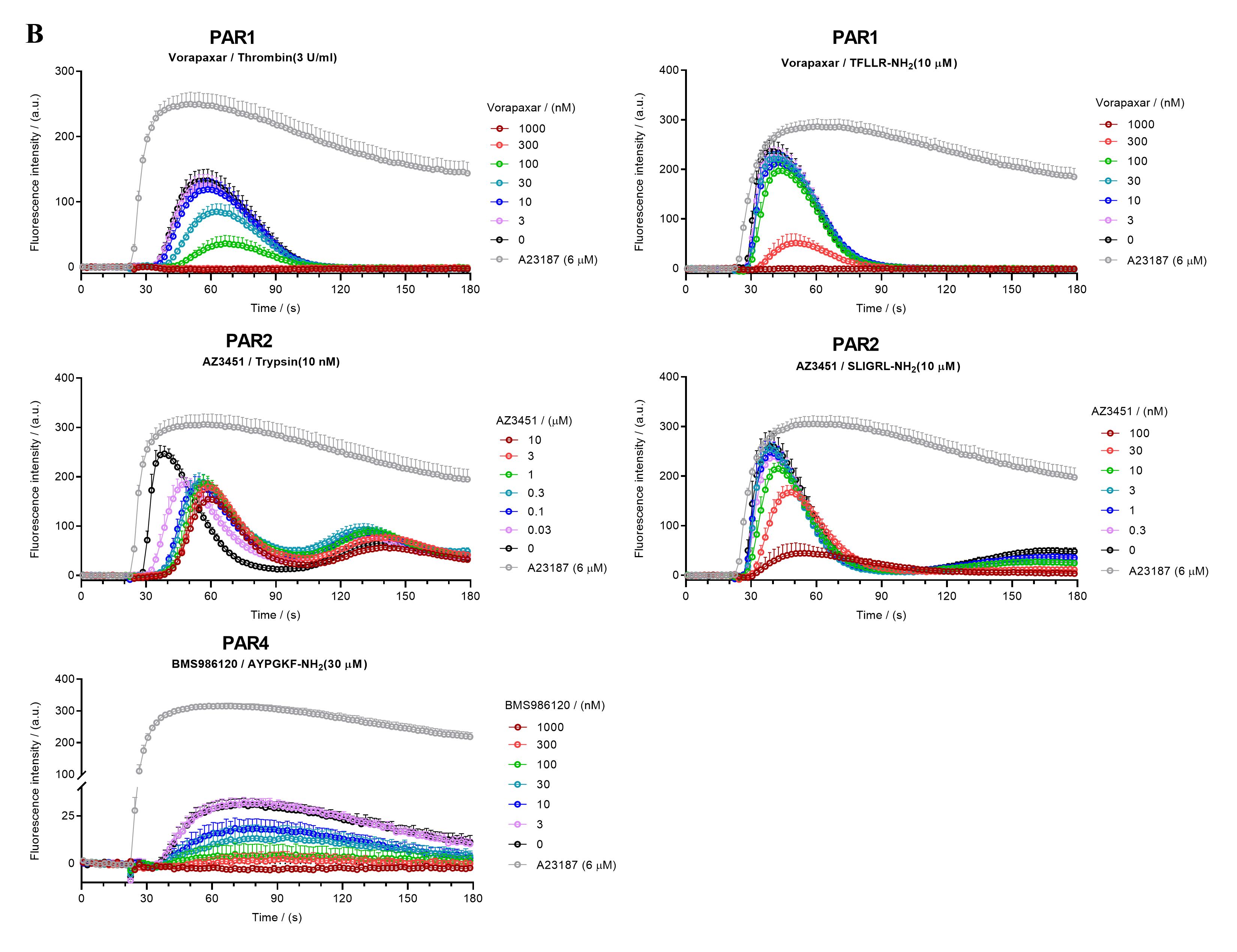
**

**Figure S4:** Representative traces for PAR1, PAR2 and PAR4 activation mediated calcium signaling. (**A**) Responses to PAR agonists, thrombin (PAR1 and PAR4), trypsin (PAR2), TFLLR-NH_2_ (PAR1), SLIGRL-NH_2_ (PAR2), and AYPGKF-NH_2_ (PAR4), and with (**B**) Responses of PAR agonists in the presence of PAR antagonists, vorapaxar (PAR1), AZ3451 (PAR2) and BMS-986120 (PAR4).

**
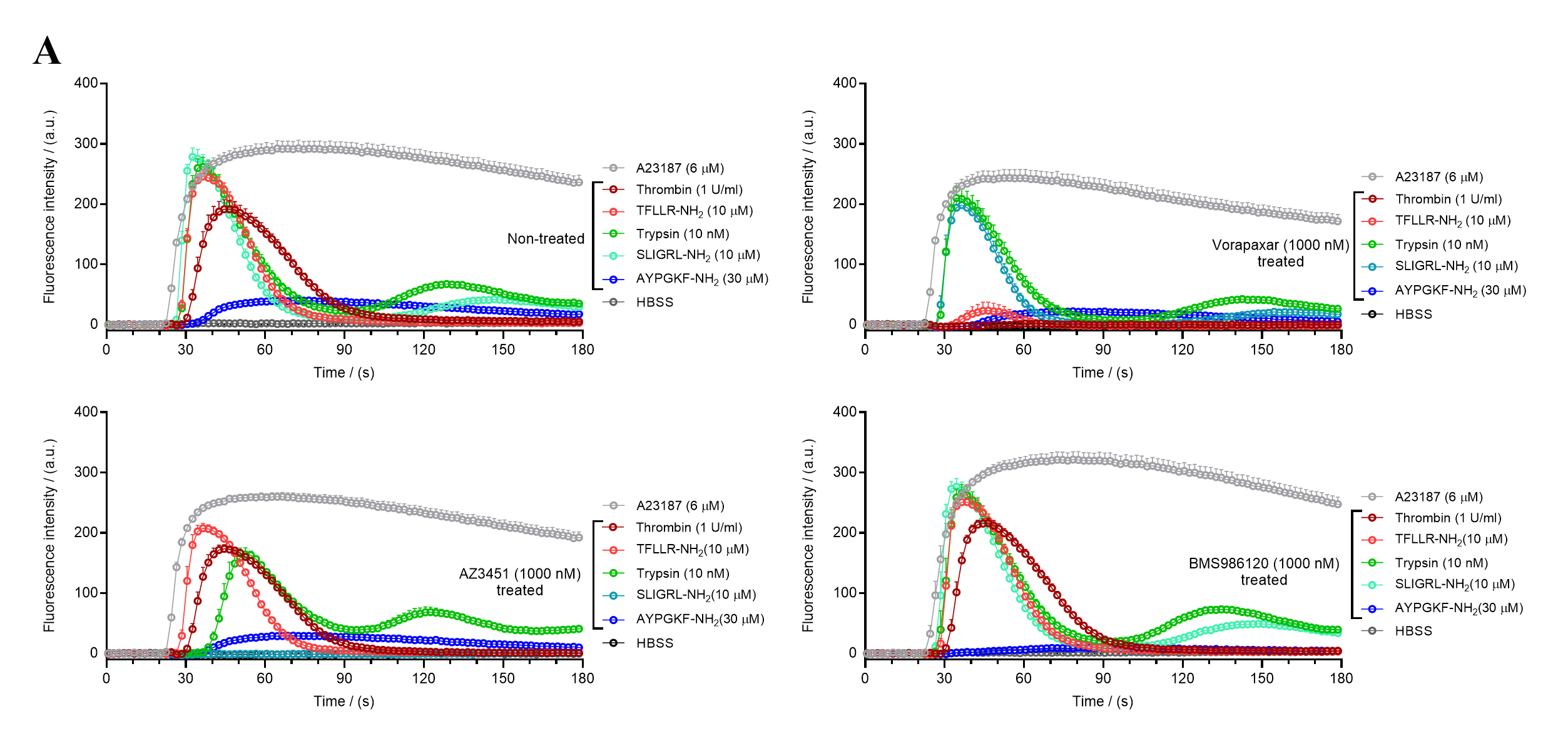
**

**
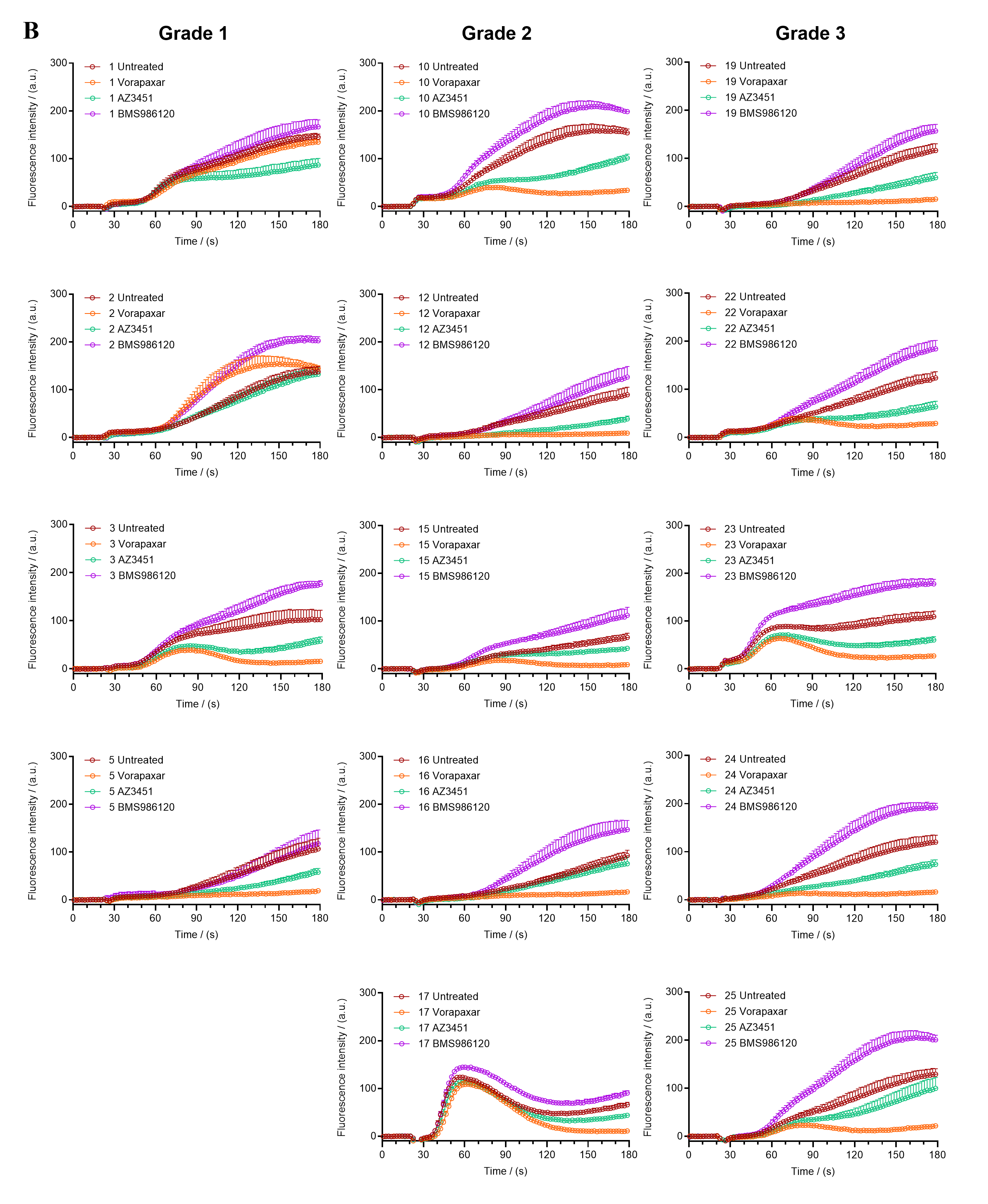
**

**Figure S5:** Calcium signaling traces of PAR activation by OA patient synovial fluids (KL grade 1-3). (**A**) Responses of controls used in the experiment, and (**B**) responses of fourteen OA synovial fluids (10%) mediated signaling in the absence and presence of PAR1, PAR2 and PAR4 antagonists. The data represent the mean ± SEM of three independent experiments (*N* = 3).

**
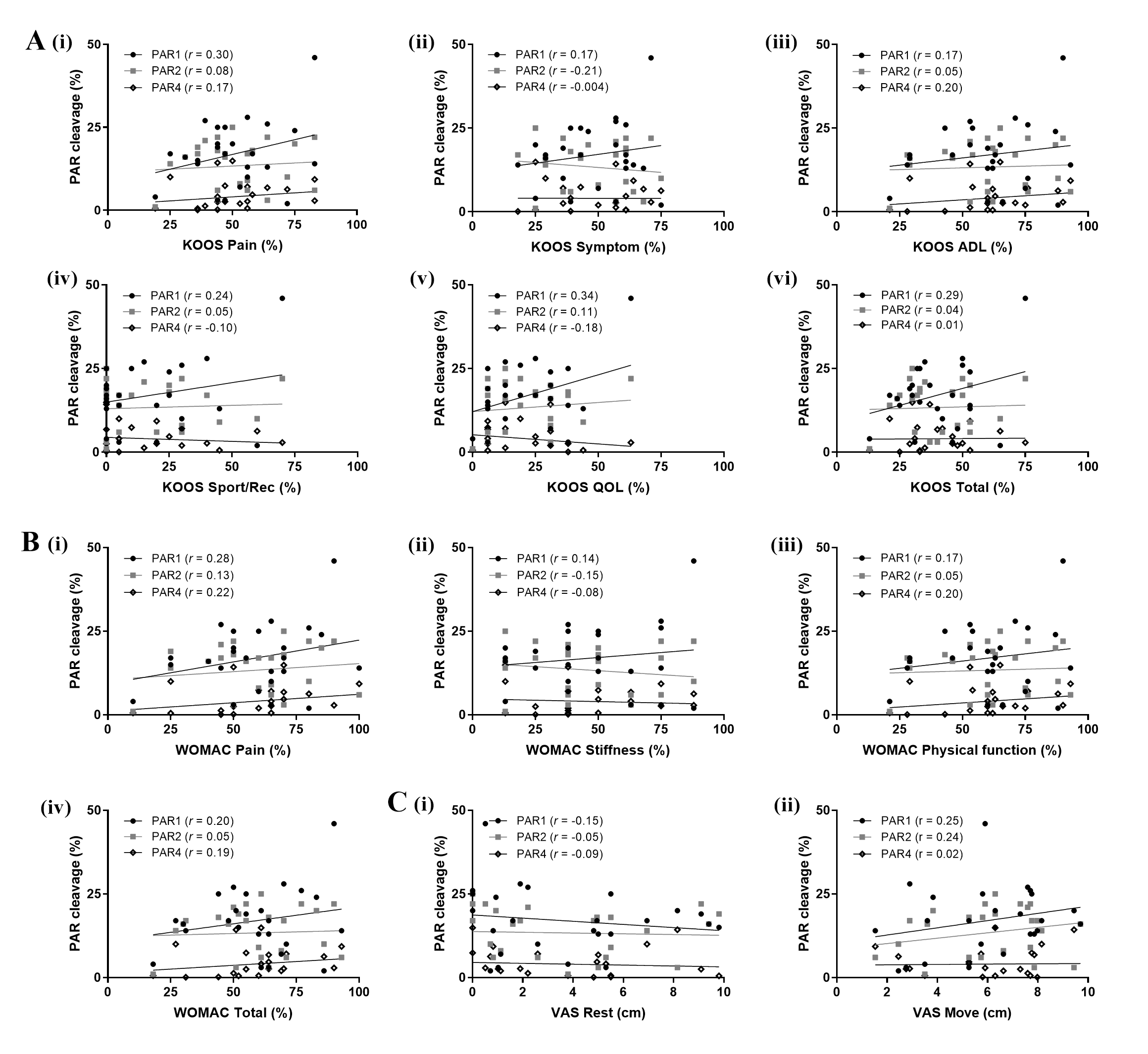
**

**Figure S6.** Correlation of the KOOS, WOMAC and VAS scores of twenty-five OA patients against the PAR1/2/4 activity. (**A**) KOOS, (**B**) WOMAC and (**C**) VAS against PARs. Pearson correlation coefficient analysis was performed to determine any correlation between PAR activity and the scores. Correlation coefficient interpretation was based on the following scale, *r* = ±1.00 to ±0.90: a very strong correlation, *r* = ±0.89 to ±0.70: a strong correlation, *r* = ±0.69 to ±0.40: a moderate correlation, *r* = ±0.39 to ±0.10: a weak correlation, and *r* = ±0.10 to ±0.00: negligible correlation.
